# *Toxoplasma gondii* infection induces extracellular vesicle miRNAs in synaptic plasticity and neural mechanisms

**DOI:** 10.20517/evcna.2025.34

**Published:** 2026-01-09

**Authors:** Poppy Cairney, Elton Rosas de Vasconcelos, Glenn A. McConkey

**Affiliations:** ^1^School of Biology, Faculty of Biological Sciences, University of Leeds, Leeds LS2 9JT, United Kingdom.; ^2^Faculty of Biological Sciences, LeedsOmics, University of Leeds, Leeds LS2 9JT, United Kingdom.

**Keywords:** Pathogen, infectious disease, neuronal, brain, noncoding RNA, dopamine, neurotransmission, exosome

## Abstract

**Aim:** This study investigates the change in profiles of miRNAs in extracellular vesicles released during *Toxoplasma gondii* *(T. gondii)* infection. *T. gondii* has been implicated in host behavioural modifications and neuroinflammatory responses, yet the molecular mechanisms involved in these changes remain poorly understood. Extracellular vesicles, involved in intercellular communication, play an important role in host-pathogen interactions, particularly through the transfer of microRNAs (miRNAs); however, the impact of extracellular vesicle miRNAs in *T. gondii* infection remains largely unexplored.

**Methods:** Human BE(2)-M17 neuronal cells were infected with Toxoplasma gondii to investigate infection-induced changes in extracellular vesicle (EV) miRNA content. EVs from infected and control cultures were isolated, characterised, and subjected to miRNA extraction followed by next-generation sequencing and differential expression analysis using standard bioinformatic pipelines. Predicted miRNA targets were integrated across multiple databases and analysed for enriched pathways to identify neuronal regulatory networks.

**Results:** Pathway network analysis identified key neurobiological pathways, including neuroplasticity, neurotransmission, and neuroinflammation in high-confidence miRNA targets with gene enrichment of neurotrophin and long-term depression and long-term potentiation, which may underlie parasite-induced alterations in neural function. Bioinformatic analysis of extracellular vesicle miRNA profiles from infected and uninfected neuronal cells revealed a set of miRNAs including *hsa-miR-4645-3p* with significant upregulation in response to infection.

**Conclusion:** These findings suggest that *T. gondii* modulates host neuronal processes through extracellular vesicle-mediated miRNA transfer, providing a potential mechanistic link between infection and parasite-associated cognitive and neuropsychiatric disturbances.

## INTRODUCTION

Since its identification over a century ago, *Toxoplasma gondii* (*T. gondii*), a parasite that can only propagate inside cells and belongs to the phylum Apicomplexa, alongside the malaria parasite, has been recognised as a significant pathogen with important consequences for human health^[1]^. Global seroprevalence estimates range from 20% to 80%, with variation driven by climatic, geographical, and socio-economic factors^[2]^. In immunocompetent hosts, *T. gondii* infection is typically asymptomatic or manifests as mild, self-limiting flu-like symptoms. This differs from infection in immunocompromised individuals and pregnant women, where the parasite can induce severe pathological outcomes^[3,4]^.

The parasite’s ability to evade immune surveillance and establish chronic infection, residing within neurons is central to its pathogenesis. *T. gondii* traverses the blood-brain barrier (BBB) and persists in the central nervous system (CNS), where it orchestrates a spectrum of inflammatory and structural changes^[5]^. Alterations in dopamine, norepinephrine (NE), and γ-aminobutyric acid (GABA) signalling pathways have been observed, alongside structural changes such as cytoskeletal rearrangements^[6-8]^. The underlying mechanisms remain incompletely understood but are thought to involve parasite-induced neuroinflammation and dysregulation of neurochemical pathways. Downregulation of dopamine β-hydroxylase (DBH), the enzyme responsible for converting dopamine to NE, has been observed, with reduced levels of NE^[6]^. These changes are accompanied by increased dopamine synthesis and release, further disrupting catecholaminergic balance. Hormonal dysregulation has also been observed in *T. gondii* infection^[9]^. Steroid hormones, particularly testosterone, have been implicated in modulating the host’s response to *T. gondii* infection, with varying effects across species and experimental conditions^[10]^. Singh *et al.* suggested that testosterone can influence host behaviour by modulating neural processes in the medial amygdala, leading to alterations in fear and predator aversion responses^[11]^. Additionally, prolactin has been shown to limit *T. gondii* replication and may play a protective role in infection, particularly in pregnant females, by enhancing immune responses and inhibiting parasite invasion^[12]^. 

Chronic *T. gondii*infection has striking effects on behaviour, demonstrated most clearly in rodents. Infected rats and mice lose their fear of predator odour with reversal to attraction as well as exhibiting an increase in motility and a decrease in anxiety^[13]^. Epidemiological studies have linked *T. gondii* infection to an increased risk of neuropsychiatric disorders, including schizophrenia and bipolar disorder, with seropositive individuals exhibiting up to a threefold higher risk^[14]^.

Extracellular vesicles (EVs) have emerged as critical mediators of *T. gondii*-induced alterations^[15]^. These vesicles likely facilitate intercellular communication within the CNS by transferring proteins, lipids, and nucleic acids^[16,17]^. *T. gondii* infects cells and induces the host cell to release a specialised subset of EVs, termed Toxoplasma-induced neuronal EVs (TINEVs), which modulate neurotransmitter synthesis and signalling. Tedford *et al.*^[15]^ demonstrated that TINEVs suppress DBH expression with epigenetic alterations, including DNA hypermethylation of the DBH promoter. An antisense long noncoding RNA for DBH was found present in EVs derived from *T. gondii*-infected cultures, showing that the parasite induces changes in the host cargo of EVs.

In this study, changes in small noncoding RNAs, termed microRNAs (miRNAs) released by cells infected with *T. gondii* were investigated. miRNAs are critical regulators of gene expression, influencing synaptic plasticity, learning, and memory^[18]^. Protozoan parasites are able to modify the expression of host miRNA to promote survival and favour infection^[19,20]^ with changes in miRNAs during *T. gondii* infection^[21]^. EVs derived from dendritic cells infected with *T. gondii* (Tg-DC-Exo) carry differentially enriched miRNAs, which reflect changes in host cell immunity^[22]^. These are enriched with *hsa-miR-155-5p*, which activates the nuclear factor kappa-light-chain-enhancer of activated B cells (NF-κB) pathway and inhibits *T. gondii* proliferation in macrophages^[23]^. The profile of host miRNAs differed in EVs of *T. gondii*-infected neuronal cells, implicating a mechanism in which the parasite alters host neurological pathways to promote survival and host behavioural changes.

## MATERIALS AND METHODS

### Cell culture and parasite propagation

The neuroblast cell line BE(2)-M17 (ATCC CRL-2267) as described in our earlier work was used^[15]^. BE(2)-M17 cells were maintained in a 1:1 ratio of Optimem and F12 Hams media (GIBCO Life Technology, Paisley, UK), supplemented with 10% horse serum (Life Technology, UK), 5% FBS (Life Technology, UK) and 100 units/mL penicillin-streptomycin (Sigma, Poole, UK).

Type II *T. gondii* (Prugniard) strains were grown and maintained on Human foreskin fibroblast cells (HFF, Hs27 ATCC CRL-1634). Monolayers were grown in Dulbecco’s Modified Eagle’s Medium (DMEM, GIBCO Life Technology, Paisley, UK) containing 10% foetal bovine serum (FBS) and 100 units/mL penicillin/streptomycin (Sigma, UK). Free tachyzoites were added to the media of a 75% confluent Human Foreskin Fibroblast monolayer for passaging. 

### Infection of cells

The objective was to identify changes in the miRNA cargo of host neuronal cell EVs during infection by intracellular *T. gondii*. To accomplish this, human neuroblastoma cells were infected and EVs produced by the infected cultures were collected for analysis. Free tachyzoites were released from human foreskin fibroblast cells by passing through a 27-gauge needle repeatedly, resuspended in 8.4 pH DMEM and incubated overnight at 37 °C to induce differentiation to bradyzoites once within cells as previously described^[15]^. Parasites were then pelleted via centrifugation (1,000 ×*g*) and alkaline media removed. BE(2)-M17 cells were seeded at a density of 5 × 10^4^/mL. At 24 h post-seeding, cells were infected with shocked parasites at a multiplicity of infection (MOI) of 1. Cells and parasites were grown in standard conditions (5% CO_2_ and 37 °C). Cell and parasite development was monitored daily using light microscopy (× 10 magnification). On day 4, the culture medium was replaced with ‘exosome-free’ FBS (GIBCO Life Technologies, Paisley, UK). On day 5, the media from infected and uninfected cultures was harvested for EV isolation. 

### Extracellular vesicle isolation and characterisation

EVs were isolated and purified using the Total Exosome Isolation Reagent (from cell culture media) (Cat No. 4478359, Invitrogen, ThermoFisher Scientific, UK) according to the manufacturer’s instructions. The cell culture media from uninfected and five-day infected cultures were initially filtered with a sterile 0.2 micron syringe filter, PVDF membrane, ThermoFisher, Cat no F2500-6 and centrifuged at 3,000 ×*g* for 30 min at 4 °C to remove cells, parasites and larger particles. One-half volume of exosome isolation reagent was added to the clarified cell culture supernatant to induce polymer-based precipitation by reduction of EV solubility with sequestration of water molecules promoting EV aggregation. Samples were incubated at 4 °C overnight, followed by centrifugation at 10,000 ×*g* for 1 h at 4 °C. The pellet was resuspended in one-tenth volume of phosphate-buffered saline (PBS) (ThermoFisher Cat no 10010023) and stored at -80 °C until analysis or use in experiments.

ZetaView®, 2nd generation (Particle Metrix GmbH) Nanoparticle Tracking Analysis (NTA) profiling service by Cell Guidance Systems (Cambridge, UK). Measurements were carried out at 24 °C in PBS (pH 7.0). Size distribution and concentration were analysed using the instrument’s size distribution mode across multiple cell positions, with quality filtering applied to remove low-confidence tracks. Key parameters including median particle size (X50), distribution span (X90-X10), and particle concentration (particles/mL) were determined using ZetaView software.

Protein concentration of purified EVs under hypotonic lysis was quantified using the Bradford assay (Bio-Rad Protein Assay Kit I Cat no 5000001, USA). Absorbance was measured at 595 nm using a spectrophotometer [UltroSpec 2100 Pro (GE Amersham), Biochrom, Cambridge, UK], with bovine serum albumin (BSA; ThermoFisher Cat no 23210; Promega, WI, USA) used to generate a standard curve.

### EV treatment of cells and target gene expression

M17 cells plated on 6-well plates and grown to a density of 2.5 × 10^4^ cells/mL were treated with purified EVs at a 10:1 culture media concentration equivalent (i.e., 1 mL of M17 cell culture treated with EVs purified from 10 mL of culture). Cells were treated for 48 h with EVs and RNA was harvested 24 h after the last treatment. Cells were pelleted at 750 ×*g* at 12 °C by centrifugation and resuspended in QIAzol reagent, and RNA isolation and purification were performed according to the manufacturer’s instructions for the Direct-zol Microprep Kit (Zymo Research). The concentration of isolated RNA was quantified using the Qubit 4 RNA High Sensitivity Assay (Cat No. Q32852, ThermoFisher Scientific, UK). Complementary DNA (cDNA) was synthesised using the SuperScript III First-Strand Synthesis Kit (Invitrogen, ThermoFisher, MA, USA) following the manufacturer’s instructions, then stored at -20 °C.

Quantitative reverse transcription polymerase chain reaction (RT-qPCR) was performed using SYBR green master mix (Life Technologies, USA) as per the manufacturer’s instructions and run using a CFX Max Real Time PCR machine (Bio-Rad, USA). Parameters were 95 °C for 2 min and followed by 40 cycles of 95 °C for 15 s, 57 °C for 15 s, and 72 °C for 45 s^[19]^. Primer sequences are recorded in Supplementary Data. The Ct value for each target gene was normalised by subtracting the Ct value of human GAPDH. The ΔCt method was used to calculate relative gene expression, with reference to the corresponding untreated control. A meltcurve was performed to check for expected products. All RT-qPCR reactions were performed with 9 replicates. The differences and variability between the expression level averages were measured using the Student’s *t*-test in GraphPad Prism 9 software. 

### miRNA extraction

miRNA was isolated from purified EV samples obtained from both infected and uninfected neuronal cell cultures using the miRNeasy kit (Cat No. 217084, Qiagen, Netherlands) in accordance with the manufacturer’s instructions. Briefly, the EVs were lysed by homogenising with buffer provided and RNA purified by affinity to a spin column and eluted with RNase-free water. The concentration of isolated miRNA was quantified using the Qubit 4 RNA High Sensitivity Assay.

### Next generation sequencing

Sequencing was performed at the Leeds Genomics Facility, a service providing library preparation and sequencing at St James’s University Hospital, Leeds. Libraries were prepared using New England Biolabs NEBNext Small RNA Library Prep Kit. Enrichment PCR was done for 15 cycles. All libraries were cleaned with the Monarch PCR and DNA Clean-up Kit, checked on Bioanalyzer High Sensitivity DNA chips, and then size-selected using BluePippin 3% gels to remove adapter dimers. All successful libraries were pooled at 0.8 ng each and quantified on the Qubit HS dsDNA kit, Bioanalyzer High Sensitivity chip and qPCR with the NEBNext Quant for Illumina kit. Pool was sequenced on the NextSeq2000 P2 100-cycle SBS chemistry kit, using a loading concentration of 550 pM and 20% PhiX spike-in. The processed samples included 6 miRNA samples (3 control, 3 infected) derived from EVs of the BE(2)-M17 cell line.

Raw sequencing reads in File and Sequencing Tools Quality (FASTQ) format were first assessed for quality using Fast Quality Control (FastQC). Adapter trimming and quality filtering were performed using Trim Galore! (v0.6.6), with default settings for small RNA sequencing. Quality reports were aggregated using Multi Quality Control (MultiQC) to ensure consistency across samples. Trimmed reads were aligned to a reference miRNA database (*mature.fa*, obtained from miRBase v22.1: https://www.mirbase.org/) using Burrows-Wheeler Aligner (BWA) (v0.7.17) with default parameters. Alignment files in sequence alignment/map (SAM) format were converted to binary alignment/map (BAM) format using SAMtools (v1.9), followed by sorting and indexing. Read counts for each miRNA were obtained using the featureCounts tool from the Subread package (v2.0.1). Raw read counts were normalised using the Transcripts Per Million (TPM) method to account for differences in sequencing depth and miRNA length across samples.

### Differential expression analysis

Raw read counts were used to assess differential miRNA expression between EVs from infected and uninfected cultures. For initial ranking, miRNAs were sorted by log2 fold change in summed TPM values across conditions, and the top 25 candidates with Log2 fold change (log2FC) > 4 were selected for further analysis. To statistically evaluate differential expression, DESeq2 was applied to the raw count matrix, with infection status as the experimental variable. miRNAs with a nominal *P*-value < 0.1 were considered for downstream interpretation. To visualise differential expression, scatter plots were generated with ggplot2 (v3.3.6). Additionally, heatmaps of top miRNAs (ranked by total read count) were constructed using pheatmap (v1.0.12) to illustrate expression patterns of log10(raw read counts) across conditions. Differentially expressed miRNA target genes were analysed further using Gene Set Enrichment Analysis (GSEA), Kyoto Encyclopedia of Genes and Genomes (KEGG), Pathview, and NETWORK via ShinyGo (v0.82)^[24-26]^.

### Functional target enrichment analysis

Differentially expressed miRNAs were subjected to target gene prediction using three established tools: miRDB (http://mirdb.org), TargetScan, and DIANA-microT-CDS (http://diana.imis.athena-innovation.gr). For miRDB, only target genes with a target score > 90 were retained^[27]^. For TargetScan (Human v8.0), predictions were filtered to include only those with a cumulative weighted context++ score ≤ -0.6, ensuring higher stringency^[28]^. DIANA-microT-CDS predictions were restricted to targets with an interaction score > 0.9^[29]^. The lists of target genes from each tool were then intersected to identify common high-confidence targets. These intersected targets were analysed using KEGG pathway enrichment via the ShinyGO platform (v0.82) to identify significantly enriched biological pathways [false discovery rate (FDR) < 0.05]^[26]^. Pathways relevant to neuronal function and infection were prioritised for interpretation.

### Statistical analysis

Statistical significance was assessed using standard thresholds for adjusted *p*-values (FDR < 0.05). All computational analyses were performed using R (v4.1.2) and Bioconductor packages.

## RESULTS

### Extracellular vesicles miRNA profiling and differential expression analysis

EVs are key mediators of intercellular communication during infection, facilitating the transfer of biomolecules such as proteins, lipids, and small RNAs^[30]^. For this study, the miRNA cargo in human neuronal cell EVs that differs when the cells are infected was analysed. NTA of EVs from *T. gondii*-infected and control cultures was performed. The analysis identified a median diameter detected of 110 nm (with 91.9 and 193.9 for 25% and 75% quartiles, respectively) for uninfected cells and median diameter detected of 130 nm (with 69 and 144 for 25% and 75% quartiles, respectively) for *T. gondii*-infected cells, consistent with the size range for exosomes [Supplementary Figure 1]^[31]^. The EV concentrations in infected and uninfected cells were similar: 2.4 × 10^9^ particles/ml from parasite-induced and 1.9 × 10^9^ particles/ml in uninfected cultures. The EVs have canonical protein markers and were visualised by transmission electron microscopy as previously^[15]^ (data not shown). EV RNA was extracted and processed for sequencing, following the analytical pipeline outlined in Supplementary Figure 1.

**Figure 1:**
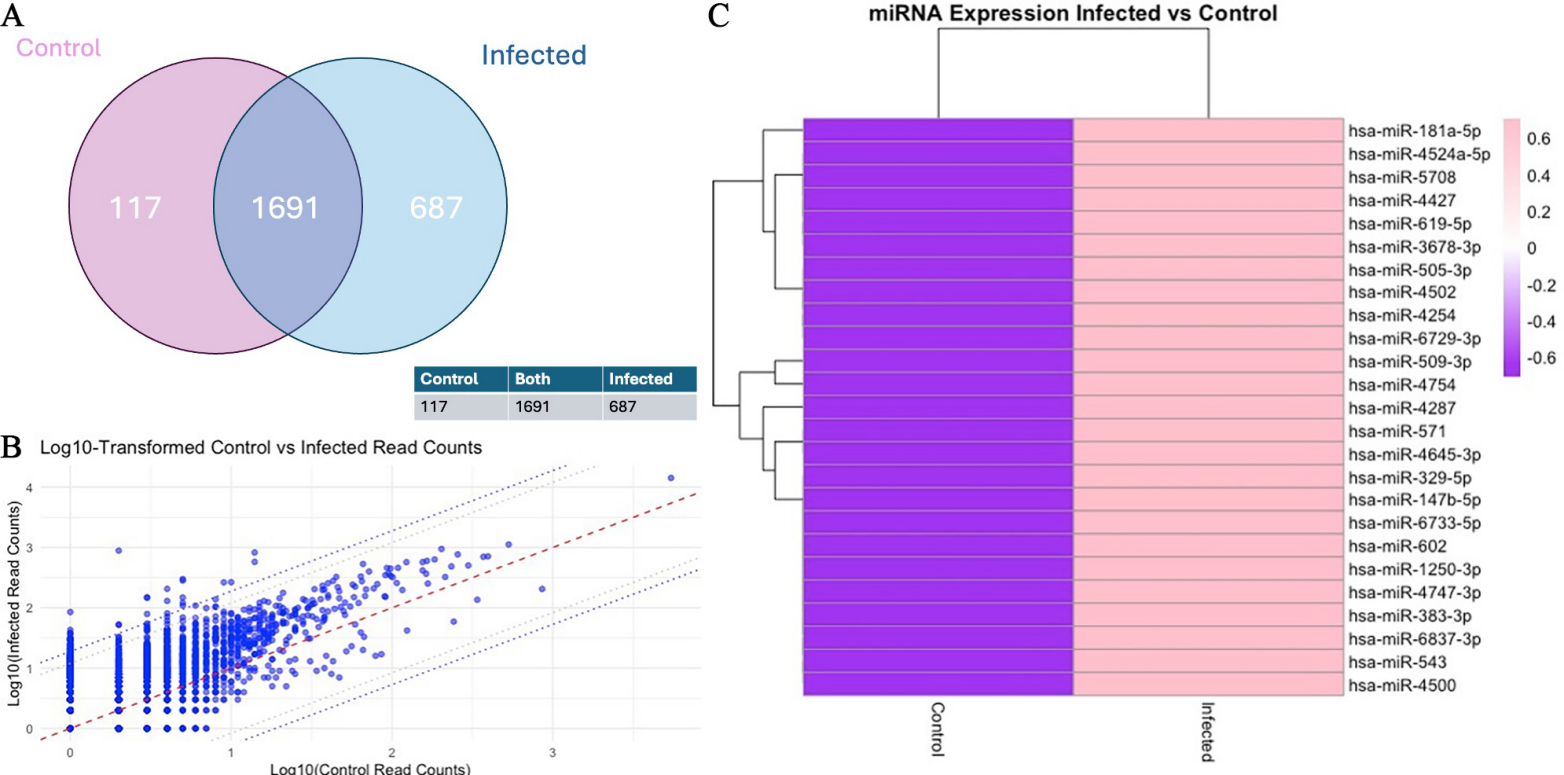
Differential miRNAs in extracellular vesicles from *T. gondii*-infected human neuronal cells. (A) Venn diagram of the read counts (TPM) illustrating the number of miRNAs that overlap between control and infected; (B) Scatter plot comparing the log10-transformed read counts (TPM) of control (x-axis) and infected (y-axis) samples for each individual miRNA. The central red dotted line represents the identity line (y = x), while the two parallel dotted lines indicate the 95th and 98th percentiles of differential expression. This plot highlights the upregulation and downregulation of miRNAs in infected samples compared to controls; (C) Heatmap of Z-score normalised log10-transformed read counts (TPM) for control and infected samples, illustrating differential expression across miRNAs. The colour scale ranges from purple (-0.6) to pink (0.6), representing the magnitude of expression changes. Hierarchical clustering was applied to both rows (miRNAs) and columns (samples) to identify groups with similar expression patterns, with branches (dendrograms) displaying these clusters. Generated using software in R.

Alignment of the sequencing reads confirmed the presence of 2,513 miRNAs in the EV RNA samples based on differential expression analysis of the three infected and three uninfected cultures. The findings of the EV miRNAs identified in infected and uninfected cultures are shown graphically in Figure 1A. Of the greater than 2,500 miRNAs identified, 687 were increased with infection and 117 were decreased in abundance with infection. To identify over- and under-abundant outlier miRNAs, expression levels were directly compared between EVs from uninfected and infected cells. This is depicted graphically [Figure 1B] with miRNAs with the 95th and 98th percentiles of differentially expressed miRNAs outside the dotted lines. The miRNAs above the 98th percentile of differential expression between control and infected samples were selected, yielding 51 candidates. These were ranked, and the top 25 miRNAs with the highest read counts were visualised in a heat map, demonstrating substantial differences in abundance between control and infected conditions [Figure 1C and Supplementary Materials]. This indicates a marked shift in the EV miRNA profile following infection.

### Target gene prediction and pathway enrichment analysis

To investigate the potential biological impact of the differentially expressed miRNAs [Table 1], predicted target genes were identified using 3 prediction tools: miRDB, TargetScan and DIANA-microT. DESeq2 *P*-value cutoff of 0.1 was applied to select targets of the top 13 overabundant miRNAs, generating a gene set for functional analysis. Pathway enrichment was performed using the KEGG database via ShinyGO^[24-26]^, identifying significantly enriched pathways with a FDR cutoff of *P* < 0.05^[26]^. 

**Table 1:** Top 25 increased miRNAs based on Log2FC

**miRNA**	***P*-value**	**Log2FC**
hsa-miR-619-5p	3.72E-05	8.79
hsa-miR-4254	0.000628	5.88
hsa-miR-6729-3p	0.00535	5.37
hsa-miR-571	0.00589	5.91
hsa-miR-4502	0.00718	5.82
hsa-miR-147b-5p	0.0114	4.95
hsa-miR-4645-3p	0.0162	4.73
hsa-miR-4524a-5p	0.0183	5.13
hsa-miR-4287	0.0443	5.69
hsa-miR-543	0.0728	4.87
hsa-miR-509-3p	0.0744	4.89
hsa-miR-4754	0.0750	4.45
hsa-miR-329-5p	0.0753	4.95
hsa-miR-181a-5p	0.119	5.64
hsa-miR-6733-5p	0.120	5.25
hsa-miR-602	0.151	5.25
hsa-miR-4500	0.171	5.60
hsa-miR-6837-3p	0.229	5.02
hsa-miR-505-3p	0.329	4.60
hsa-miR-4747-3p	0.369	4.73
hsa-miR-1250-3p	0.519	5.43
hsa-miR-4427	0.627	4.72
hsa-miR-3678-3p	0.845	4.34
hsa-miR-5708	0.850	4.70
hsa-miR-383-3p	0.941	4.34

Log2FC: Log2 fold change.

Table 1 lists the top 25 miRNAs showing the greatest overabundance from infected-cell samples relative to uninfected control conditions, from 51 outlier miRNAs that exceeded the 98th percentile in differential read counts. Log2FC values greater than 4 indicate substantial upregulation in infected samples. miRNAs are ranked by ascending *P*-value.

### Pathway analysis of upregulated miRNAs

**Figure 2:**
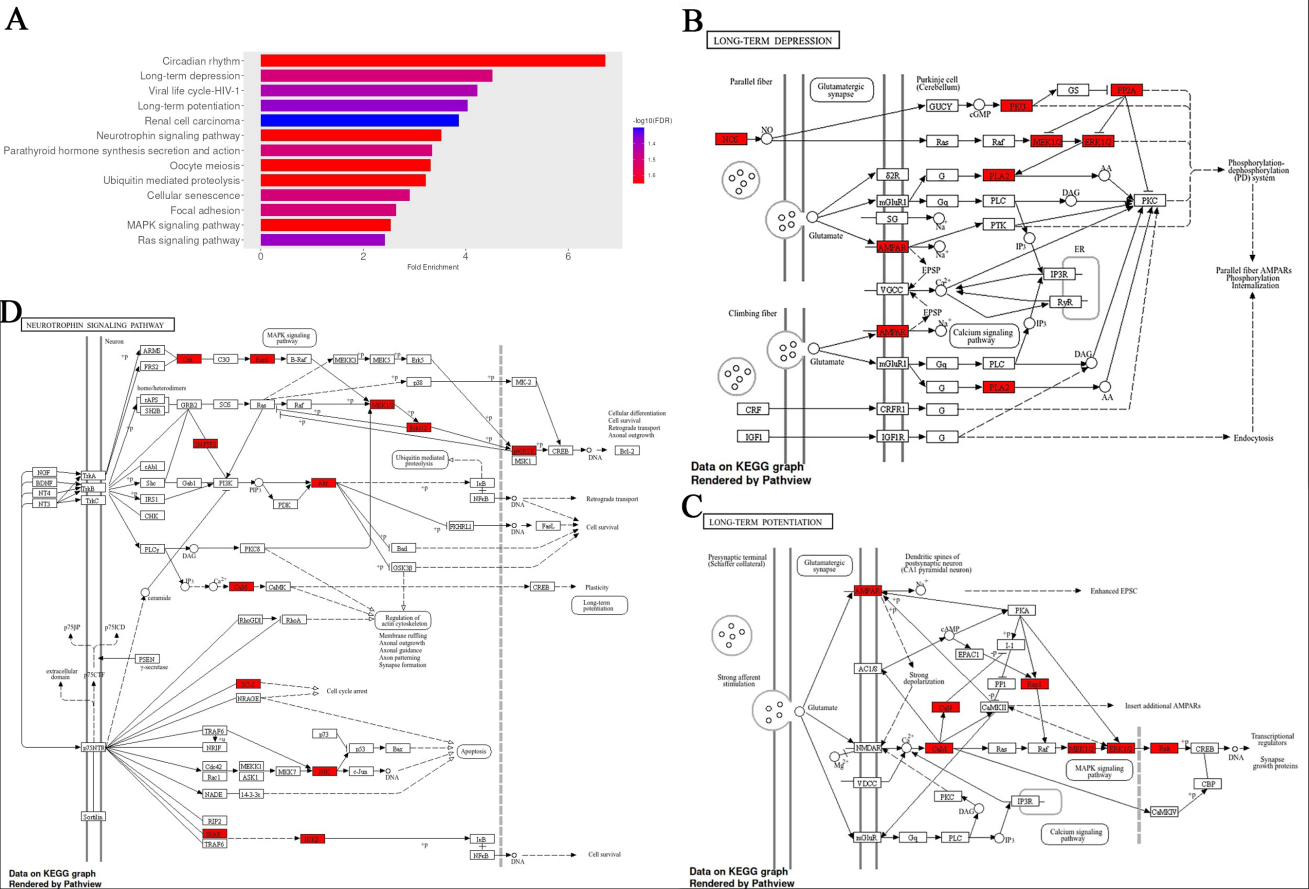
Fold enrichment analysis of pathways associated with genes that are targets of top the 13 upregulated EV miRNAs with infection. (A) Fold enrichment chart for biological processes, horizontal bars represent the magnitude of enrichment for each pathway, calculated as fold enrichment relative to background expectation. Colour intensity reflects statistical significance, with a gradient based on -log_10_(FDR); darker blue tones indicate lower false discovery rates and thus stronger enrichment confidence; (B-D) KEGG pathway analysis for the long-term depression, long-term potentiation, and Neurotrophin signalling, respectively, with enrichment results using an FDR cutoff of 0.05. Red-colored genes represent those linked to the top 13 differentially expressed miRNAs. All figures were generated using the ShinyGO platform v0.82. Full size images of A-D, and MAPK & Ras signalling pathways are shown in the Supplementary Materials. EV: Extracellular vesicle; KEGG: Kyoto Encyclopedia of Genes and Genomes; FDR: false discovery rate; Ras: rat sarcoma.

Pathway enrichment was performed using the top-scoring predicted target genes for each of the 13 most overabundant miRNAs (*P* < 0.1; Supplementary Materials). Enriched pathways were prioritised based on fold enrichment and FDR, with pathways involving calcium signalling being among the top-ranked [long-term depression (LTD), long-term potentiation (LTP), neurotrophin signalling, oocyte meiosis, cellular senescence, rat sarcoma (Ras) signalling] [Figure 2]. Circadian rhythm showed the highest level of enrichment, consistent with its established role in maintaining cellular homeostasis^[32]^. LTP, a fundamental process underlying synaptic plasticity, learning, and memory, is critically dependent on calcium influx through N-methyl-D-aspartate (NMDA) receptors and the subsequent activation of calcium/calmodulin-dependent kinases and mitogen-activated protein kinase (MAPK) signalling cascades^[33]^. The enrichment of LTP-related genes therefore aligns with the observed prominence of calcium signalling pathways. In addition, there were high ranking pathways associated with brain ageing and neurodegeneration, neurogenesis, and adrenergic signalling, such as neurotrophin, MAPK, and Ras signalling^[34-36]^.

### Functional analysis of the overabundant miRNA targeting calmodulin

**Figure 3:**
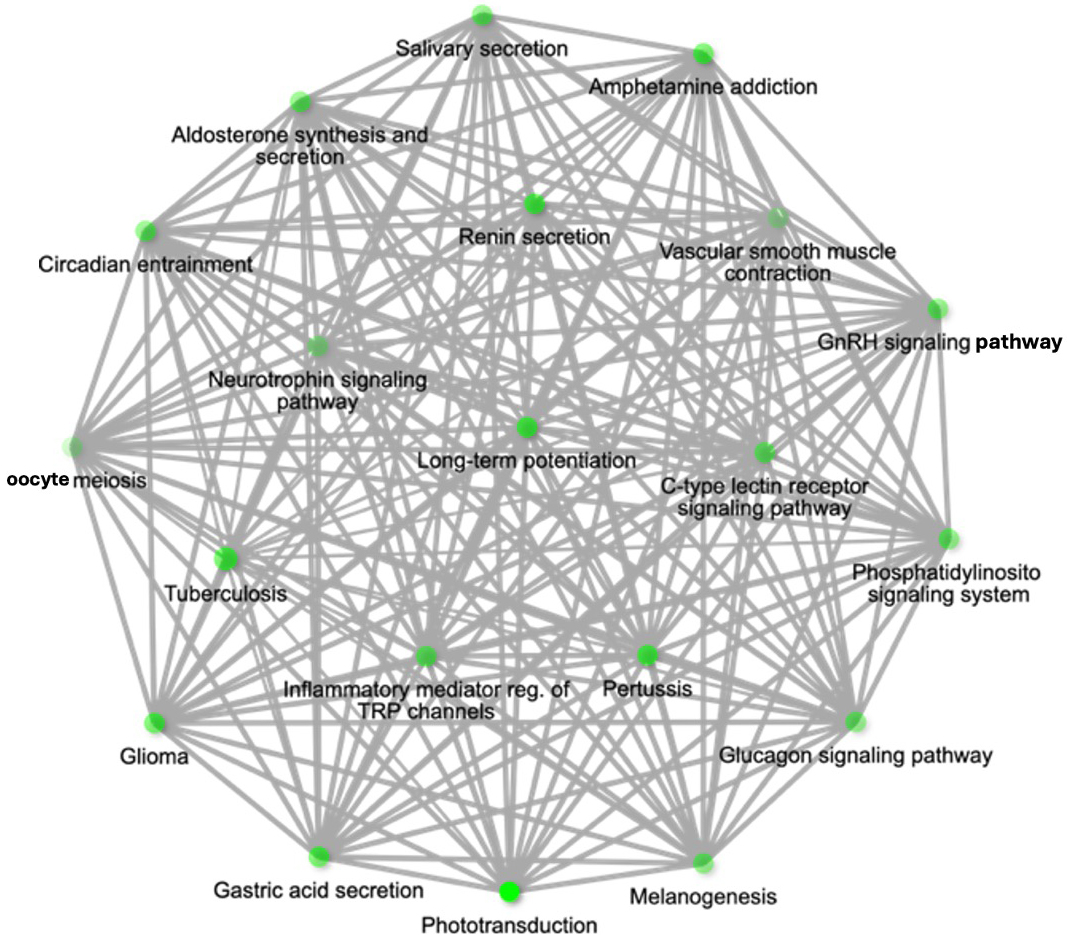
Pathway network for upregulated miR-4645-3p and associated pathways of *hsa-miR-4645-3p* represented as a hierarchical clustering tree. Pathway network diagram showing the target genes of *hsa-miR-4645-3p* within threshold with 3 prediction tools. The network highlights the relationship between enriched pathways, where 2 nodes (pathways) are connected if they share 20% or more genes. Darker nodes represent more significantly enriched gene sets, while larger nodes indicate larger gene sets. Thicker edges between nodes denote greater overlap of genes between pathways. The pathways are enriched based on the target genes of miR-4645-3p.

During pathway analysis, calcium signalling was a repeated theme in over-represented pathways linked to EV miRNAs overabundant with *T. gondii* infection. Consistent in these was miRNA *hsa-miR-4645-3p*. Hence, it emerged as a candidate of interest due to its strong association with pathways central to the focus of this study [Figure 3]. 

The behavioural-linked pathways most significantly associated with *hsa-miR-4645-3p* included LTP (Fold Enrichment = 64, FDR = 1.3 × 10^-4^), the Neurotrophin signalling pathway (Fold Enrichment = 36.1, FDR = 2.3 × 10^-4^), and the Gonadotropin-releasing hormone GnRH signalling pathway (Fold Enrichment = 46.1, FDR = 1.6 × 10^-4^) [Figure 3B]. 

Calmodulin (CaM), an important protein in calcium signalling, appeared in multiple enriched pathways such as LTP, Neurotrophin signalling and Ras signalling. *CALM2,* which encodes calmodulin 2, a crucial mediator of calcium signal transduction^[37]^, was selected for further investigation due to the role calcium signalling and calmodulin play in *T. gondii *tachyzoite proliferation, invasion and egress of neuronal cells^[38]^. *hsa-miR-4645-3p* is closely associated with CALM2, with a target score of 99 out of 100 for CALM2 on the miRDB webserver, an interaction score of 0.99 for CALM2 on the DIANA-microT webserver, and CALM2 identified as the leading target (supported by 22,519 3P-seq tags) based on 14 features, which are comparable to the best high-throughput *in vivo* crosslinking approaches on TargetScan^[28]^. The miRNA hsa-miR-4645-3p has binding sites at 91 and 158 bases in the 3’ untranslated region (UTR). 

**Figure 4:**
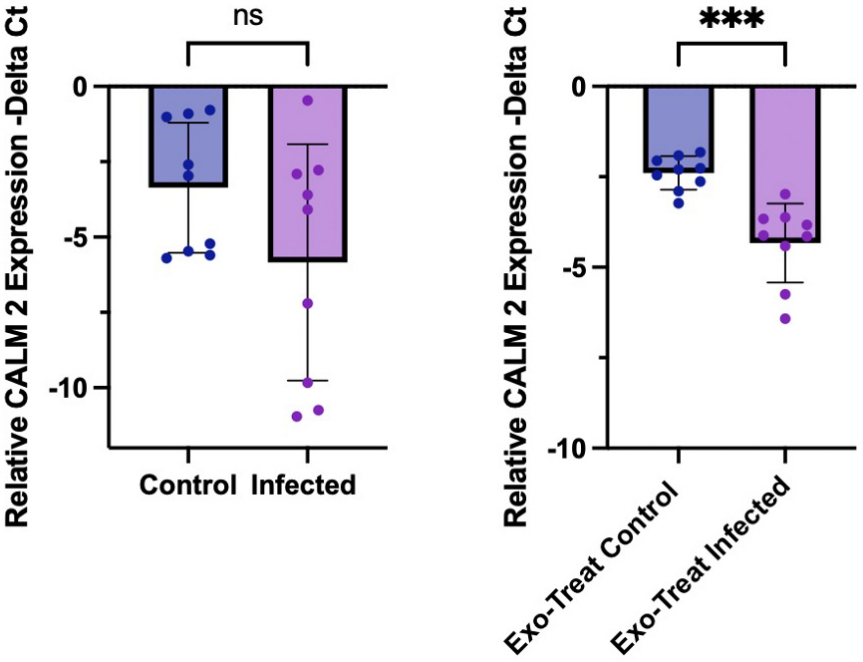
Quantitative PCR analysis of CALM2 expression. Relative messenger RNA (mRNA) levels of *CALM2* (encoding calmodulin 2) were quantified by qPCR and normalised to *GAPDH* expression, presented as -ΔCt values. (A) Expression levels in uninfected and *T. gondii*-infected M17 cell cultures; (B) Expression in M17 cells treated with EVs, showing a significant reduction under infected conditions. Statistical analysis was performed using Welch’s *t*-test in GraphPad Prism. Error bars indicate standard deviation; *P* < 0.05. PCR: Polymerase chain reaction; EV: extracellular vesicle.

Consistent with the pathway enrichment findings, quantitative reverse transcription polymerase chain reaction (RT-PCR) analysis revealed a significant downregulation of *CALM2* expression in EV-treated M17 cells (*P* = 0.0005), indicating that EV miRNA cargo may contribute to the suppression of host calcium signalling pathways [Figure 4]. Although *CALM2* expression also trended downward in directly infected cells, the variability observed between replicates may reflect heterogeneity in parasite differentiation states, specifically incomplete transition from tachyzoite to bradyzoite forms resulting in variable levels of host cell stress, apoptosis, and survival. The more consistent and statistically significant downregulation in the exosome-treated condition supports that EVs, rather than direct infection alone, mediate a targeted post-transcriptional repression of *CALM2*.

## DISCUSSION

In this study, we found that *T. gondii* infection of human neuronal cells, with catecholaminergic and dendritic properties, results in a distinct profile of EV miRNAs. A collection of human miRNAs was increased in EVs derived from infected cells [Table 1]. The overabundant miRNAs can modulate gene expression in cells exposed to these EVs. This can have major effects on cell function, from response to infection to signalling involved in animal behaviour.

Pathway enrichment analysis of predicted targets from the top overabundant miRNAs revealed significant association with several neurobiologically-relevant pathways, including LTP, LTD and Neurotrophin signalling. These pathways are central to the regulation of neuronal excitability, synaptic plasticity, and behavioural adaptation. The prominence of these pathways indicates that infection-induced EVs may modulate fundamental processes underlying neuronal communication and plasticity responsible for observed changes in behaviour with *T. gondii *infection. LTP and LTD represent complementary mechanisms of synaptic modification that together regulate excitatory signalling and underlie adaptive learning and memory processes^[39-41]^. The involvement of calcium-dependent signalling and calmodulin-regulated kinases within these pathways further supports a role for EV-associated miRNAs in influencing intracellular calcium dynamics and downstream transcriptional regulation. The concurrent enrichment of Neurotrophin, MAPK, and Ras signalling pathways points to broader effects on neuronal differentiation, stress responsiveness, and synaptic remodelling. Dysregulation of these signalling axes has been reported during *T. gondii* infection, consistent with parasite-mediated interference in host cell signalling cascades^[42]^. Choopani *et al.* observed that *T. gondii* infection can impair learning and memory through altered hippocampal LTP and synaptic plasticity**^[43]^**. Infection-driven changes in neurotrophin signalling and synaptic marker expression further implicate these pathways in the neural response to infection^[44]^. The enrichment of these pathways highlights that EVs released from *T. gondii*-infected neuronal cells may exert multifaceted effects on host neural function through miRNA manipulation. 

The miRNA among those in the enriched pathways with a greater than twenty-fold increase in read count in EVs from infected cultures was *hsa-miR-4645-3p*. *T. gondii* infection resulted in 26.6-fold increased levels of *hsa-miR-4645-3p* in EV cargo. The leading target of *hsa-miR-4645-3p*, the calmodulin 2 gene (CALM2), encodes calmodulin whose role in neurons is sensing Ca^2+^ levels in synaptic plasticity and release of neurotransmitters. For example, facilitating NE synaptic vesicle fusion. Furthermore, plasticity is essential in learning and memory, two processes that have been found impaired by chronic *T. gondii* infection^[43,45]^. As expected with this elevation in *hsa-miR-4645-3p*, expression of the target gene CALM2 was down-regulated with exposure to TINEVs [Figure 4]. 

Predicted targets of *hsa-miR-4645-3p *revealed strong associations with key neurobiological pathways, including LTP, GnRH signalling, and Neurotrophin signalling. Homeostatic plasticity, particularly through synaptic scaling, allows neurons to balance their excitatory and inhibitory synaptic strengths, creating a negative feedback mechanism to stabilise activity levels during prolonged changes^[15,46]^. Decreased LTP may have behavioural consequences during infection. Prior studies have shown that *T. gondii*-infected rodents exhibit enhanced risk-taking and altered responses to predator cues^[47]^, risk-taking behaviours that are thought to stem from neuroplasticity changes in limbic structures such as the amygdala and hippocampus^[48]^. Dopaminergic and noradrenergic signalling are intricately involved with LTP, risk-taking and memory; all functions that are altered during chronic infection with *T. gondii*^[49]^. *T. gondii* infection dysregulated dopamine release and NE has been established with links to a parasite-derived tyrosine hydroxylase^[50]^. The enrichment of neurotrophin signalling further supports the asserted role of signalling, as neurotrophins, particularly brain-derived neurotrophin factor (BDNF), are critical regulators of synaptic function and neuronal survival^[51]^. Dysregulation of this pathway has been implicated in both neurodevelopmental and neurodegenerative disorders, suggesting that parasite-induced perturbations may have long-term consequences on host neural circuitry^[49]^. GnRH, as a key regulator of reproductive and neuroendocrine functions, raises the possibility that EV miRNA transfer contributes to the hormonal and behavioural changes observed in infected individuals^[52]^. Hormonal imbalances have been observed during *T. gondii* infection with several studies on testosterone^[53]^. This study suggests a mechanism for the observed modulation of neuroendocrine pathways via miRNA released in EVs from *T. gondii*-infected cells.

Inflammatory responses to infection were also identified by enrichment analysis including mediating regulation of Transient Receptor Potential (TRP) channels and phosphatidylinositol signalling [Figure 3]. The former is particularly relevant given that TRP channels are central players in neuroinflammatory responses and sensory transduction, and these should be the subject of future studies of chronic parasitic infections^[54,55]^ as well as neuroinflammation as previously reported^[9,56,57]^.

This study is limited by the use of an established neuronal cell line although *ex vivo* human neuron samples are ethically restricted. A larger number of samples could have been analysed. The target identification of miRNAs relied on predictions in three miRNA databases. The study is further limited by demonstrating differential expression of only the target of miRNA *hsa-miR-4645-3p* CALM2 which exhibited down-regulation confirming this target; similar analyses could be expanded to other miRNAs. The modulation of pathways associated with targets of the miRNAs needs verification in future studies.

Although these findings provide compelling evidence for the role of EV miRNAs in *T. gondii* pathogenesis, several questions remain. Firstly, while pathway analysis offers insights into potential functional consequences, direct validation of further miRNA-mRNA interactions is needed in future studies. Further, future research will differentiate EVs originating from infected cells and investigate the mechanisms for sorting the EV miRNAs, for eventual application in development of treatments. Additionally, the downstream phenotypic effects of EV-mediated miRNA transfer warrant further investigation. Given that *T. gondii* can persist in the CNS for extended periods^[5]^, understanding whether these miRNAs contribute to long-term neurophysiological alterations could have important implications for neuropsychiatric and neurodegenerative disease research. This study identifies a distinct EV miRNA signature associated with *T. gondii* infection in human neuronal cells.

## DECLARATIONS

### Availability of data and materials

All data generated and analysed during this study are included. The raw data supporting the findings of this study are available within this Article and its Supplementary Materials. Further data are available from the corresponding authors upon request.

### Copyright

© The Author(s) 2026.
