# Supplementary material for: Toxoplasma gondii infection induces extracellular vesicle miRNAs in synaptic plasticity and neural mechanisms
Source: Extracell Vesicles Circ Nucl Acids. 2026 Jan 9;7(1):1–15. doi: 10.20517/evcna.2025.34 (PMC13074276; doi:10.20517/evcna.2025.34)

Manuscript Title: ***Toxoplasma gondii*** **infection induces extracellular vesicle miRNAs in synaptic plasticity and neural mechanisms**

Manuscript Author:

Poppy Cairney[1], Elton Rosas de Vasconcelos[2], and Glenn A. McConkey[1]

[1]School of Biology, Faculty of Biological Sciences, University of Leeds, Leeds LS2 9JT, United Kingdom.

[2]Faculty of Biological Sciences, LeedsOmics, University of Leeds, Leeds LS2 9JT, United Kingdom.

Correspondence to: Dr. Glenn McConkey, School of Biology, University of Leeds, Leeds LS2 9JT, United Kingdom. E-mail: G.A.McConkey@Leeds.ac.uk

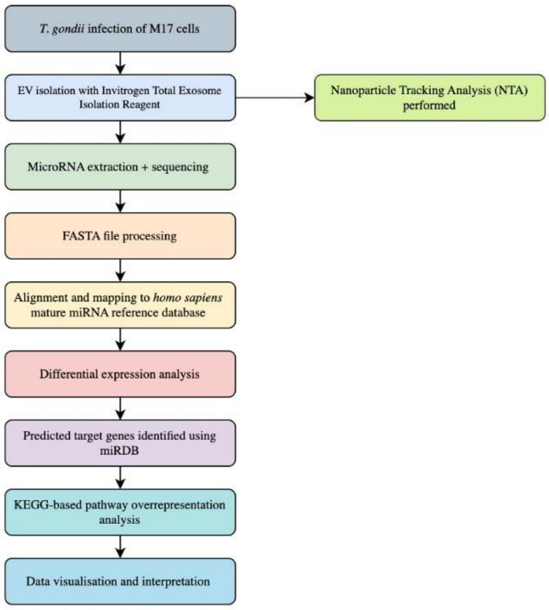

Supplementary Figure 1. Workflow for miRNA-Seq Data Processing and Analysis. This flowchart outlines the stepwise pipeline used for miRNA sequencing analysis. BE(2)-M17 cells were cultured, with half the samples infected with Toxoplasma gondii (1:1 MOI). Nanoparticle Tracking Analysis (NTA) was performed to confirm EV size and concentration. Small RNA was extracted (n=6), followed by library preparation and sequencing. The raw sequencing data underwent preprocessing, quality control, and alignment to the Homo sapiens miRNA reference database (mature.fa). Read counts were obtained using featureCounts, and differential expression analysis was performed using DEseq2 package in

R. The p-value cutoff applied was 0.1. Target prediction was conducted using miRDB, and KEGG-based pathway enrichment analysis was carried out using ShinyGo. Visualisation techniques, including heatmaps and scatterplots, were used to interpret differentially expressed miRNAs and their associated pathways.

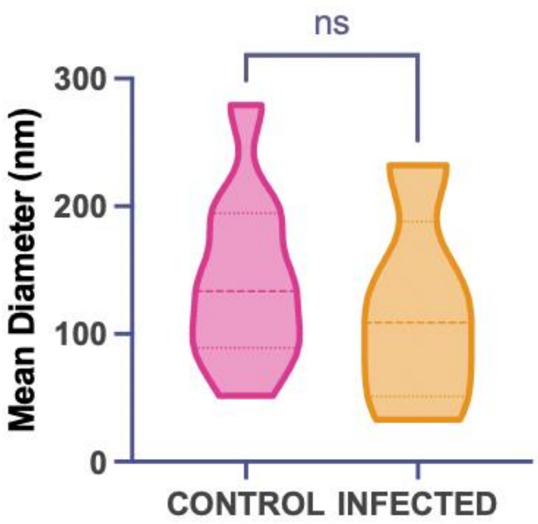

Supplementary Figure 2. *Exosomal vesicle size distribution in control and infected samples.* Violin plot showing the mean diameter (nm) of exosomes isolated from control and *Toxoplasma gondii*-infected M17 cultures, measured using nanoparticle tracking analysis (NTA). Statistical comparison was performed using an unpaired two-tailed *t*-test ($t = 0.6261$, df = 13), yielding a *p*-value of 0.5421, indicating no significant difference in vesicle size between conditions. Median and quartiles are indicated within each distribution.

Full size of **Figure 2.**

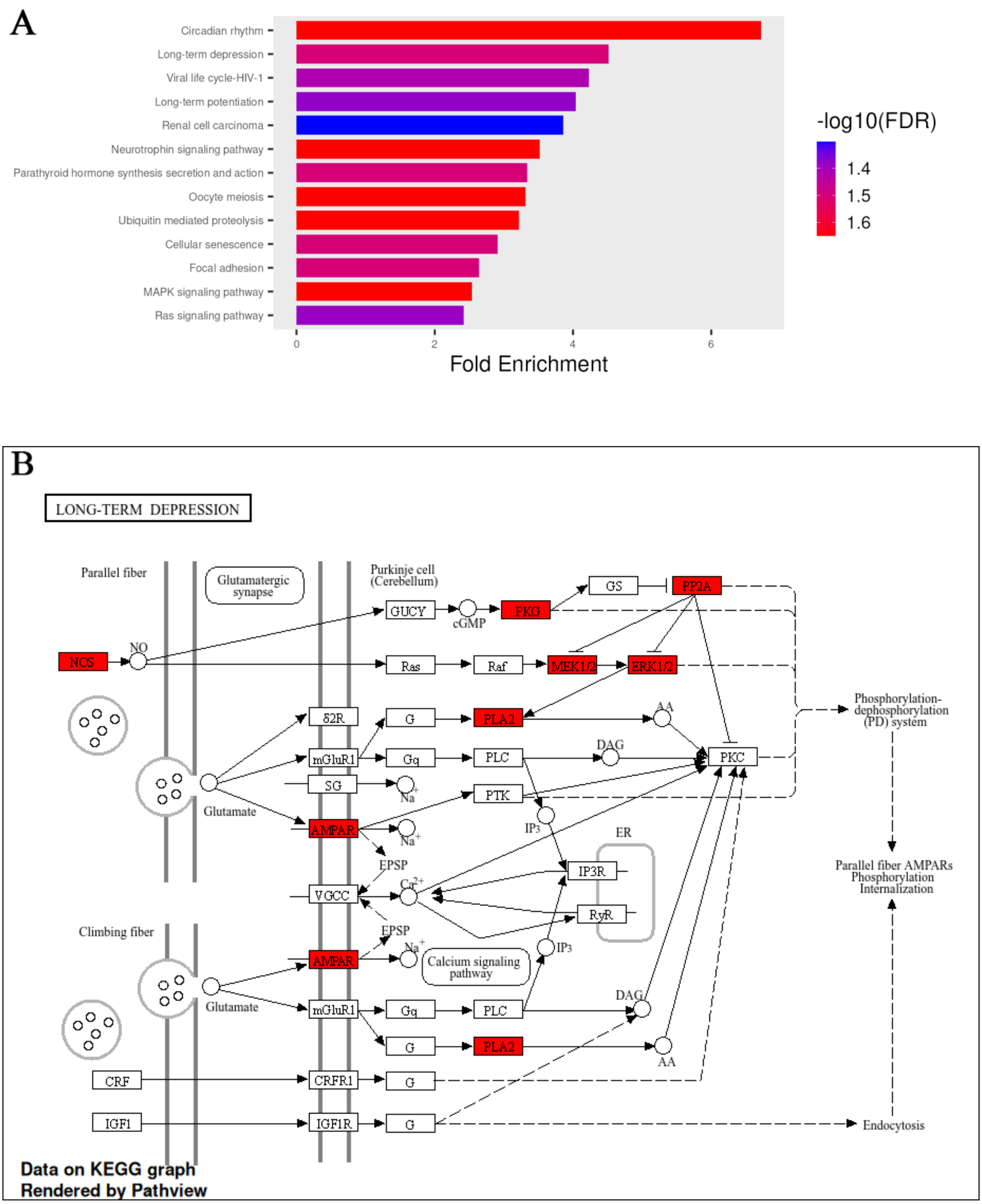
A
Circadian rhythm
Long-term depression
Viral life cycle-HIV-1
Long-term potentiation
Renal cell carcinoma
Neurotrophin signaling pathway
Parathyroid hormone synthesis secretion and action
Oocyte meiosis
Ubiquitin mediated proteolysis
Cellular senescence
Focal adhesion
MAPK signaling pathway
Ras signaling pathway
0
2
4
6
Fold Enrichment
-log10(FDR)
1.4
1.5
1.6
B
LONG-TERM DEPRESSION
Parallel fiber
Glutamatergic synapse
Purkinje cell (Cerebellum)
Climbing fiber
Glutamate
NOS
NO
GUCY
cGMP
PKG
GS
PP2A
Ras
Raf
MEK1/2
ERK1/2
δ2R
G
PLA2
AA
mGluR1
Gq
PLC
DAG
PKC
SG
Na+
PTK
AMPAR
IP3
ER
EPSP
IP3R
VGCC
Ca2+
RyR
Calcium signaling pathway
CRF
CRFR1
IGF1
IGF1R
Phosphorylation-dephosphorylation (PD) system
Parallel fiber AMPARs Phosphorylation Internalization
Endocytosis
Data on KEGG graph
Rendered by Pathview

C

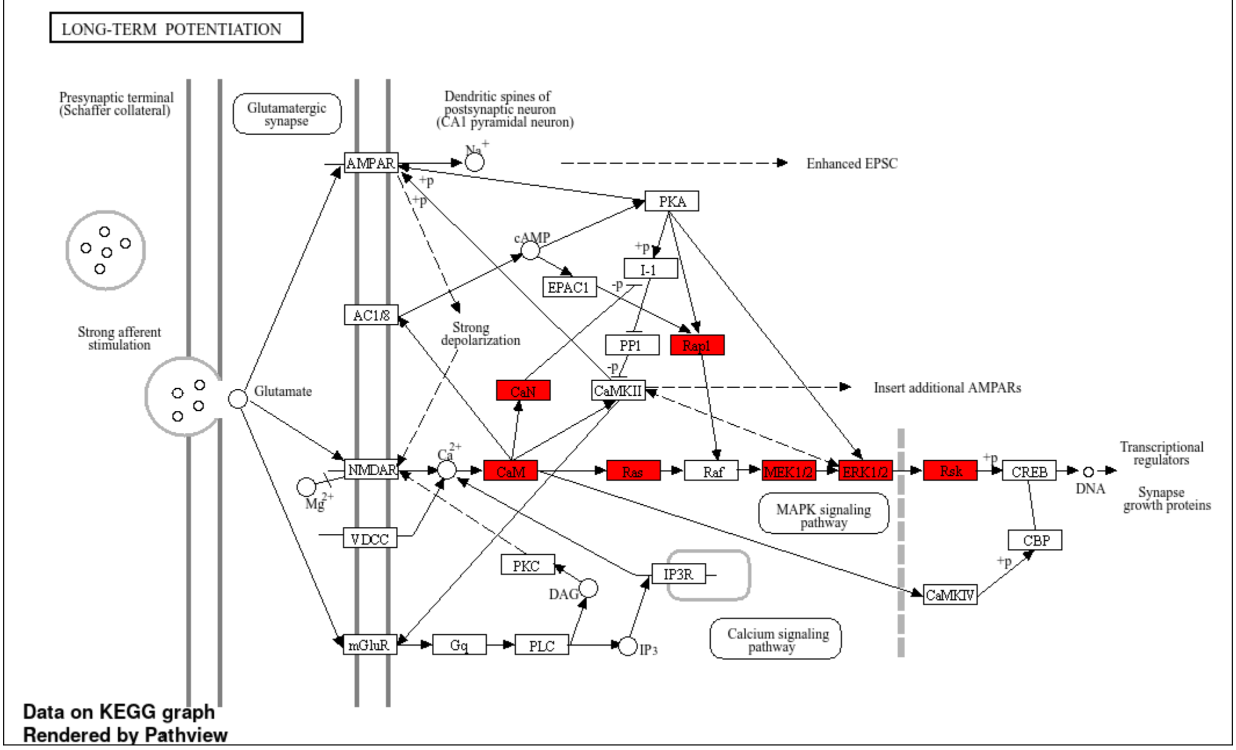

Data on KEGG graph
Rendered by Pathview

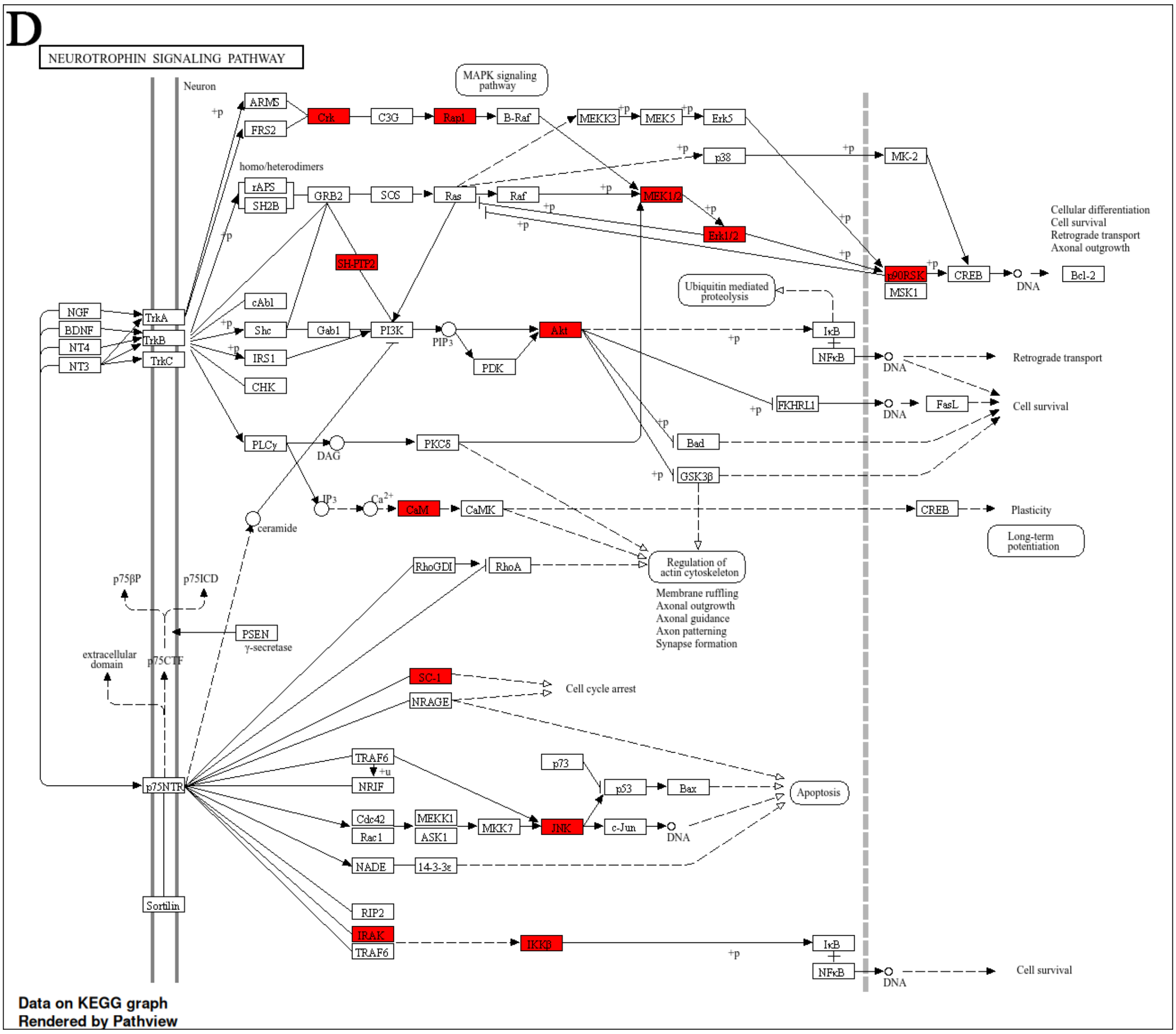
D
NEUROTROPHIN SIGNALING PATHWAY
Neuron
MAPK signaling pathway
ARMS
FRS2
Crk
C3G
Rap1
B-Raf
MEKK3
MEK5
Erk5
homo/heterodimers
rAPS
SH2B
GRB2
SOS
Ras
Raf
MEK1/2
Erk1/2
p38
MK-2
SH-PTP2
cAbl
Shc
Gab1
PI3K
PIP3
PDK
Akt
IRS1
CHK
NGF
BDNF
NT4
NT3
TrkA
TrkB
TrkC
p90RSK
MSK1
CREB
DNA
Bcl-2
Cellular differentiation
Cell survival
Retrograde transport
Axonal outgrowth
Ubiquitin mediated proteolysis
IκB
NFκB
Retrograde transport
Cell survival
FKHRL1
FasL
Bad
GSK3β
PLCγ
DAG
PKCδ
IP3
Ca2+
CaM
CaMK
CREB
Plasticity
Long-term potentiation
ceramide
Regulation of actin cytoskeleton
Membrane ruffling
Axonal outgrowth
Axonal guidance
Axon patterning
Synapse formation
RhoGDI
RhoA
p75βP
p75ICD
PSEN
γ-secretase
extracellular domain
p75CTF
SC-1
NRAGE
Cell cycle arrest
TRAF6
NRIF
p75NTR
p73
p53
Bax
Apoptosis
Cdc42
Rac1
MEKK1
ASK1
MKK7
JNK
c-Jun
NADE
14-3-3ε
Sortilin
RIP2
IRAK
TRAF6
IKKβ
Cell survival
Data on KEGG graph
Rendered by Pathview

MAPK Signaling

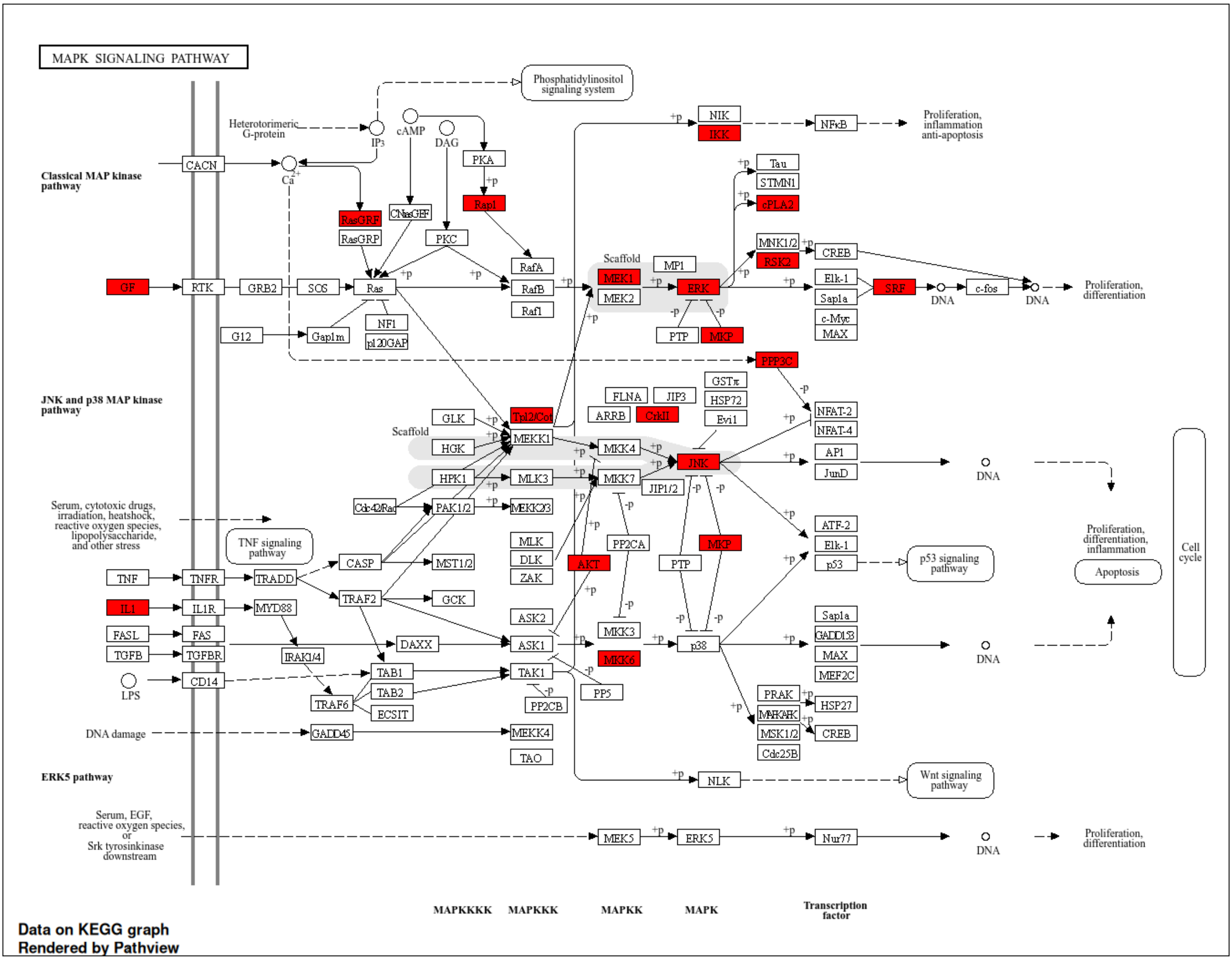

Ras signaling

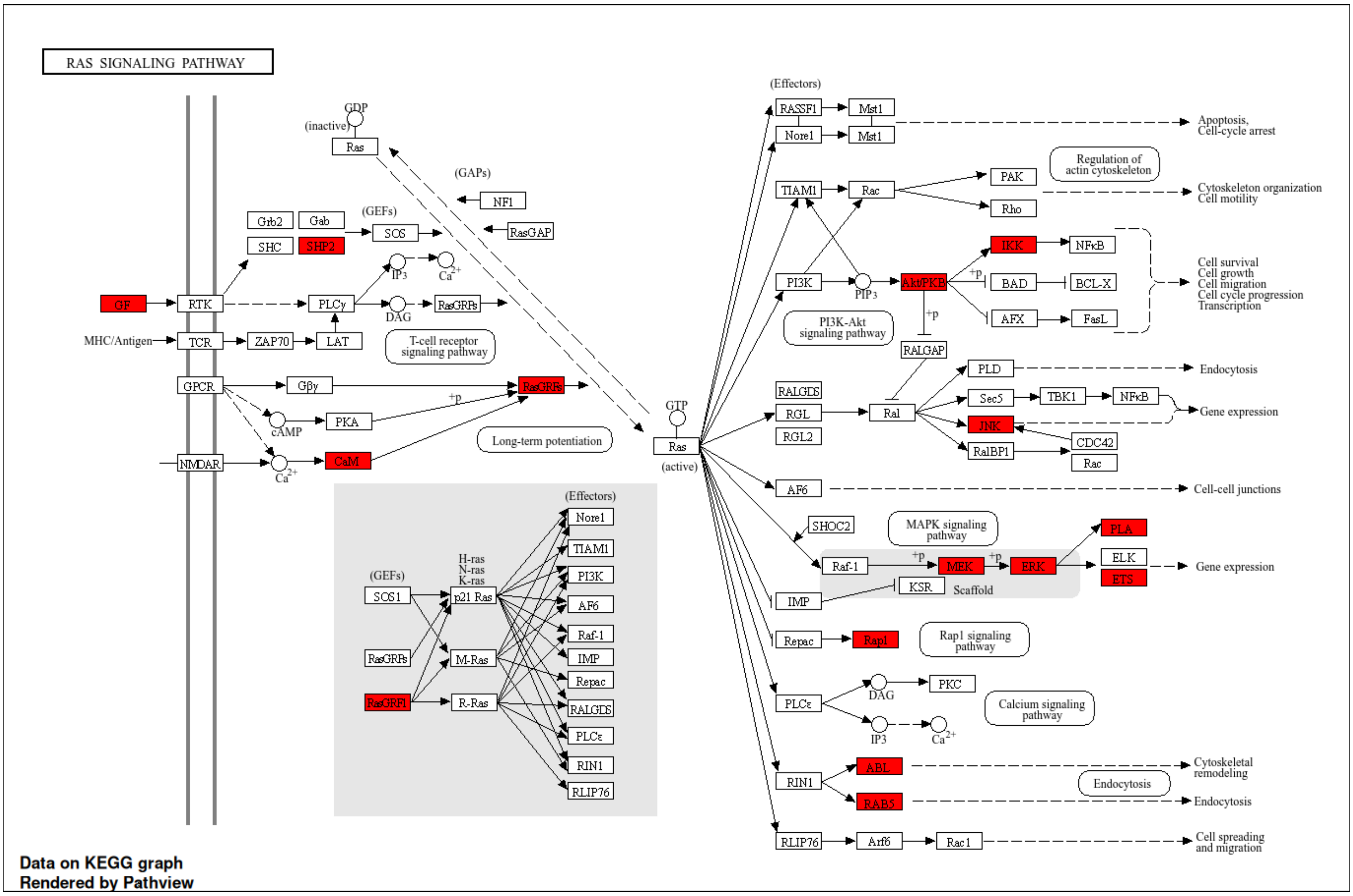

Supplement: Supplementary file 1 [file evcna-7-1-1-SupplementaryMaterials.zip › evcna6034-SupplementaryMaterials.pdf]
